# Tissue plasminogen activator is a ligand of cation-independent mannose 6-phosphate receptor and consists of glycoforms that contain mannose 6-phosphate

**DOI:** 10.1038/s41598-021-87579-z

**Published:** 2021-04-15

**Authors:** James J. Miller, Richard N. Bohnsack, Linda J. Olson, Mayumi Ishihara, Kazuhiro Aoki, Michael Tiemeyer, Nancy M. Dahms

**Affiliations:** 1 Department of Biochemistry, Medical College of Wisconsin, 8701 W. Watertown Plank Rd, Milwaukee, WI 53226 USA; 2 Complex Carbohydrate Research Center, University of Georgia, 315 Riverbend Rd, Athens, GA 30602 USA

**Keywords:** Biochemistry, Cell biology

## Abstract

Plasmin is the key enzyme in fibrinolysis. Upon interaction with plasminogen activators, the zymogen plasminogen is converted to active plasmin. Some studies indicate plasminogen activation is regulated by cation-independent mannose 6-phosphate receptor (CI-MPR), a protein that facilitates lysosomal enzyme trafficking and insulin-like growth factor 2 downregulation. Plasminogen regulation may be accomplished by CI-MPR binding to plasminogen or urokinase plasminogen activator receptor. We asked whether other members of the plasminogen activation system, such as tissue plasminogen activator (tPA), also interact with CI-MPR. Because tPA is a glycoprotein with three N-linked glycosylation sites, we hypothesized that tPA contains mannose 6-phosphate (M6P) and binds CI-MPR in a M6P-dependent manner. Using surface plasmon resonance, we found that two sources of tPA bound the extracellular region of human and bovine CI-MPR with low-mid nanomolar affinities. Binding was partially inhibited with phosphatase treatment or M6P. Subsequent studies revealed that the five N-terminal domains of CI-MPR were sufficient for tPA binding, and this interaction was also partially mediated by M6P. The three glycosylation sites of tPA were analyzed by mass spectrometry, and glycoforms containing M6P and M6P-*N*-acetylglucosamine were identified at position N448 of tPA. In summary, we found that tPA contains M6P and is a CI-MPR ligand.

## Introduction

The plasminogen activation system degrades fibrin clots and extracellular matrix components, which allows for cell migration during blood vessel repair. Some cancer cells take advantage of this system as a method of metastasis (reviewed in^1,2^). Plasminogen is a zymogen that is activated to plasmin, a ~ 90 kDa serine protease that consists of seven domains: an N-terminal regulation domain, five kringle domains, and a C-terminal catalytic domain^3^. Two well-known plasminogen activators are urokinase-type plasminogen activator (uPA) and tissue plasminogen activator (tPA). tPA is a ~ 70 kDa glycoprotein with three N-linked glycosylation sites (N117, N184, and N448)^4^ and has been extensively characterized due to its clinical use in the context of ischemic strokes. tPA has also been implicated in roles other than fibrinolysis, such as in neuronal migration, learning, memory, addiction, and anxiety (reviewed in^5,6^) as well as in the degradation of amyloid-β aggregates^7^.

Cation-independent mannose 6-phosphate receptor (CI-MPR), also known as insulin-like growth factor 2 receptor (IGF2R), is a type I membrane protein with diverse functions. CI-MPR is a P-type lectin that is essential in the intracellular sorting of lysosomal enzymes modified with mannose 6-phosphate (M6P) on their N-linked glycans. The CI-MPR extracellular region consists of 15 homologous domains, where domains 3, 5, 9, and 15 bind carbohydrates, and domain 11 binds IGF2. CI-MPR is known to exist as a dimer in the membrane^8,9^. At the cell surface, CI-MPR captures secreted lysosomal enzymes by undergoing receptor-mediated endocytosis. CI-MPR also binds insulin-like growth factor 2 (IGF2), which allows for IGF2 downregulation by lysosomal degradation. Thus, CI-MPR accomplishes its well-studied functions through its multiple domains with defined ligand specificities (reviewed in^10–12^). Recent studies have shown that allosteric cooperativity is a key feature of CI-MPR’s ability to bind ligands with high affinity^13^.

In addition to lysosomal enzymes and IGF2, reports have also shown that CI-MPR directly binds some members of the plasminogen activation system, such as plasminogen and uPA receptor (uPAR)^14–22^. The binding of plasminogen and uPAR have been mapped to the N-terminal domain of CI-MPR (domain 1). However, the biological consequences of these interactions are less clear. Nykjær et al. demonstrated that CI-MPR binds uPAR in a M6P-independent manner and is capable of directing uPAR to lysosomes^19^. Similar to this study, Leksa et al. showed that CI-MPR mediates plasminogen internalization^20^. However, other studies suggest that CI-MPR acts as a scaffold at the cell surface to approximate plasminogen and uPAR, allowing for plasmin generation and subsequent activation of TGF-β^21,22^. These findings are conflicting because in one case, CI-MPR downregulates plasminogen activation, while in the other case, CI-MPR promotes plasminogen activation. Furthermore, it is unknown whether the regulatory effects of CI-MPR on the plasminogen activation system are cell type specific. For example, a recent study by Leksa’s group demonstrated that CI-MPR mediates plasminogen-induced efferocytosis^23^.

We aimed to further understand the role of CI-MPR in plasminogen activation and asked whether other members of the plasminogen activation system, such as tPA, interact with CI-MPR. Because tPA is a glycoprotein with three N-linked glycosylation sites, we hypothesized that tPA binds CI-MPR in a M6P-dependent fashion. Using surface plasmon resonance (SPR) binding assays, we found that tPA bound to the entire extracellular region of CI-MPR and the five N-terminal domains of CI-MPR with low to mid nM affinity. The binding between CI-MPR and tPA was partially inhibited by phosphatase treatment or the addition of M6P. To our knowledge, our mass spectrometric analysis revealed for the first time that tPA consists of glycoforms that contain M6P and M6P-*N*-acetylglucosamine in low abundance at N448. We identified tPA as a new CI-MPR ligand, and future studies are needed to fully elucidate the roles of CI-MPR on tPA function.

## Methods

### Materials

Two different preparations of tPA were obtained: native tPA and recombinant tPA. Native tPA, which is isolated from a human melanoma cell line, was purchased from Calbiochem (now Millipore Sigma). Recombinant human tPA was purchased from the Froedtert and Medical College of Wisconsin pharmacy as Cathflo Activase (alteplase), which is manufactured by Genentech. This recombinant version of tPA is purified from the medium of a Chinese hamster ovary (CHO) cell line engineered to express and secrete human tPA. M6P, G6P, and αMeMan were acquired from Sigma. Human β-glucuronidase was prepared as previously described^14^.

### Cloning of CI-MPR constructs

A DNA cassette encoding domains 1 through 5 (amino acids 36–763) of the human CI-MPR (CI-MPR d1-5) was amplified by PCR from the full-length ATCC clone # 95661 and ligated into a modified pVL1392 vector that encodes the bovine CI-MPR signal sequence upstream of the ligation site. The 3-prime end of the clone encodes a thrombin cleavage site followed by six histidine residues and the BirA biotinylation acceptor site (AviTag) (5′-CTTGTTCCTCGGGGATCCCACCATCACCATCACCATTCTAGAGCTCCAGGTTT GAACGATATATTTGAAGCTCAGAAAATTGAATGGCATGAA-3′). A similar cassette was also made to encode human CI-MPR domains 1 through 15 (amino acids 36–2286) (CI-MPR d1–15). Bovine CI-MPR d1–15 (amino acids 47–2296) was generated in a similar cassette but without an AviTag.

### Expression and purification of CI-MPR constructs

CI-MPR constructs were expressed in a *Spodoptera frugiperda* cell line (Sf9, Expression Systems) using serum-free medium (ESF921, Expression Systems) and purified as previously described^24^. Media containing the secreted CI-MPR constructs were dialyzed against buffer containing 20 mM Tris and 300 mM NaCl (pH 8.0). Dialyzed medium was passed over nickel-nitrilotriacetic acid-agarose resin (Clonetech), washed, and then eluted with buffer containing 20 mM Tris, 150 mM NaCl, and 400 mM imidazole (pH 8.0). Following nickel chromatography purification, the protein was concentrated and then buffer exchanged on a sizing column (Sephacryl S-300, GE Healthcare) equilibrated in 20 mM HEPES and 150 mM sodium acetate. The proteins were concentrated, and those proteins containing a C-terminal AviTag were biotinylated using BirA ligase (BirA500, Avidity) per the protocol provided by the manufacturer.

### Binding affinity studies using surface plasmon resonance

SPR measurements were performed at 25 °C using a Biacore 3000 instrument (Cytiva, formerly GE Healthcare Life Sciences). CM5 sensor chips and surfactant polysorbate-20 were obtained from Cytiva. Streptavidin (Thermo Fisher), tPA, bovine CI-MPR d1–15, or β-glucuronidase were coupled to the sensor chip surface by amine coupling as described previously^25^. Purified, biotinylated human CI-MPR d1-5 and human CI-MPR d1–15 were immobilized by passing them over the streptavidin-coupled surface in 20 mM HEPES, 150 mM sodium acetate, 0.005% polysorbate-20 (pH 7.4). Protein samples were centrifuged for 10 min at 10,000×*g* to pellet any particulates. Injected proteins (i.e., analytes) were passed over the surfaces at a flow rate of 40 µl/min for 2 min in running buffer. All response data were double-referenced as described^26^. Briefly, we controlled for refractive index changes by subtracting a reference flow cell sensorgram from all binding sensorgrams. The concentration curves were analyzed and fit with a one site specific binding model (GraphPad Prism 8):

$$ Response\; at \;Equilibrium = \frac{{B_{max} \times concentration_{tPA} }}{{K_{d} + concentration_{tPA} }} $$

### Dephosphorylation of tPA and β-glucuronidase

Proteins (10 µg) were mixed with 4 units alkaline phosphatase (FastAP, Thermo Fisher) in buffer: 10 mM Tris–HCl (pH 8.0 at 37 °C), 5 mM MgCl_2_, 100 mM KCl, 0.02% Triton X-100, and 0.1 mg/ml BSA. The mixture was incubated at 37 °C for 4 h. The reaction was quenched by the addition of β-glycerophosphate to a final concentration of 5 mM. Proteins were immediately diluted in SPR running buffer: 10 mM HEPES, 150 mM sodium acetate, 5 mM β-glycerophosphate, and 0.005% polysorbate-20 (pH 7.4). “Mock-treated” proteins were prepared in the absence of alkaline phosphatase.

### Mass spectrometry

tPA N-glycans were analyzed as described previously^13^. Briefly, recombinant and native tPA glycoproteins were subjected to acetone precipitation to get rid of contaminating detergent. Aliquots of tPA were then reduced, carboxyamidomethylated, dialyzed against nanopure water at 4 °C overnight for desalting, and then dried in a centrifugal concentrator. The dried, desalted sample was resuspended in 50 mM ammonium bicarbonate buffer and then digested with the following three protease conditions: a chymotrypsin alone, a mixture of trypsin and Glu-C, or a mixture of chymotrypsin and Glu-C (Promega). Following digestions, the samples were dried and subsequently resuspended in solvent A (0.1% formic acid in water) and passed through a 0.2 μm filter (Nanosep, PALL) before analysis by liquid chromatography-tandem mass spectrometry (LC–MS/MS). LC–MS/MS analyses were performed on an Orbitrap-Fusion or Orbitrap-Fusion Lumos equipped with an EASY nanospray source and Ultimate3000 autosampler LC system (Thermo Fisher). Resuspended peptides were chromatographed on a nano-C18 column (Acclaim pepMap RSLC, 75 μm 515 × 150 mm, C18, 2 μm) with an 80-min gradient of increasing mobile phase B (80% acetonitrile, 0.1% formic acid in distilled H_2_O) at a flow rate of 300 nl/min routed directly into the mass spectrometer. Full MS spectra were collected at 60,000 resolution in FT mode and MS/MS spectra were obtained for each precursor ion by data-dependent scans (top-speed scan, 3 s) utilizing CID, HCD, or ETD activation and subsequent detection in FT mode.

Phosphorylated glycopeptides were annotated by manual data interpretation of the LC MS/MS data following initial processing by Byonic software (Protein Metrics). Byonic parameters were set to allow 20 ppm of precursor ion monoisotopic mass tolerance and 20 ppm of fragment ion tolerance. Byonic searches were performed against the human tissue plasminogen activator (tPA) sequence allowing modification with phosphorylated and non-phosphorylated human/mammalian N-glycans. Time-averaged full MS spectra were deconvoluted for charge state by Xtract (Thermo Fisher) to facilitate relative quantification of peptide glycoforms.

## Results

### tPA binds to the extracellular region of CI-MPR

**Figure 1:**
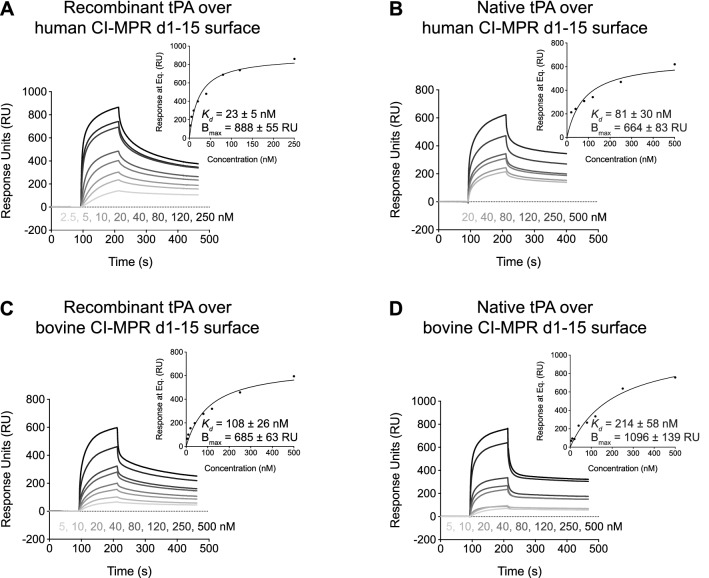
tPA from two sources binds the extracellular region of CI-MPR from two species. (**A**,**B**) Human CI-MPR d1–15 was biotinylated at the C-terminus and immobilized to a streptavidin sensor chip. Sensorgrams are shown for various concentrations of injected recombinant tPA (**A**) or various concentrations of injected native tPA (**B**). (**C**,**D**) Bovine CI-MPR d1–15 was amine coupled to a sensor chip. Sensorgrams are shown for various concentrations of injected recombinant tPA (**C**) or various concentrations of injected native tPA (**D**). In panels (**A**–**D**), the inset plots show the response at equilibrium (Eq.) with respect to tPA concentration. Data are fit using a one site specific binding fit, and *K*_*d*_ and B_max_ are reported below the curve (best fit value ± SEM). *CI-MPR d1–15* cation-independent mannose 6-phosphate receptor domains 1–15, *tPA *tissue plasminogen activator.

We performed SPR experiments to investigate the potential interaction between tPA and CI-MPR. We expressed the entire extracellular region (domains 1–15, abbreviated here as d1–15) of both human and bovine CI-MPR. Human CI-MPR d1–15 was expressed with a C-terminal AviTag, and the resulting protein was biotinylated using BirA ligase and immobilized to a streptavidin sensor chip. This method allowed for protein coupling to the chip in a uniform orientation (i.e., C-terminus coupled with N-terminus untethered). Bovine CI-MPR d1–15 was not expressed with a C-terminal AviTag and was immobilized to a sensor chip by amine coupling. Two sources of tPA were obtained: (1) recombinant tPA that was overexpressed in CHO cells, and (2) native tPA that was produced by melanoma cells. These tPA samples were injected over immobilized human or bovine CI-MPR d1–15 at various concentrations, and responses were observed (Fig. 1). Using a one site specific binding fit, the dissociation constant (*K*_*d*_) values were calculated to be 23 ± 5 nM with recombinant tPA (Fig. 1A) and 81 ± 30 nM with native tPA (Fig. 1B) when human CI-MPR was immobilized. When tPA was injected over the bovine CI-MPR d1–15 surface, the calculated *K*_*d*_ values were 108 ± 26 nM with recombinant tPA (Fig. 1C) and 214 ± 58 nM with native tPA (Fig. 1D). Therefore, two sources of tPA are able to bind the extracellular region of human and bovine CI-MPR with low to mid-nM affinity.

### A phosphorylated mannose residue on tPA partially mediates binding to CI-MPR

**Figure 2:**
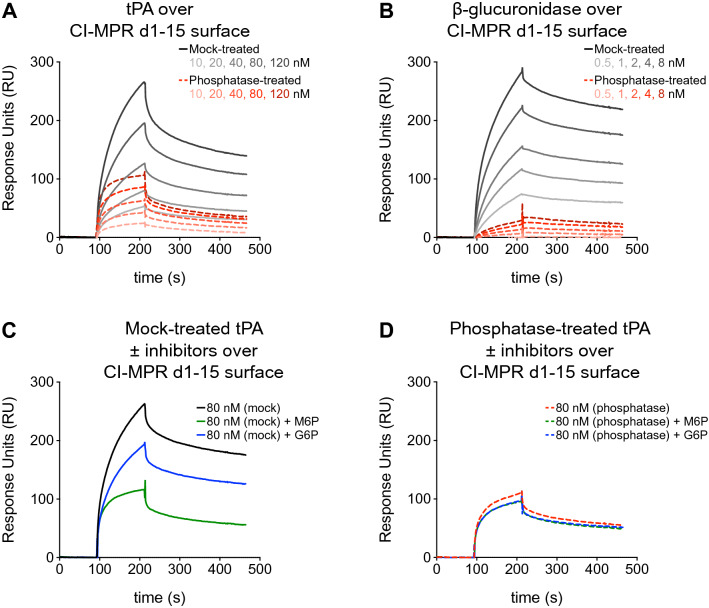
What is the nature of the interaction between tPA and CI-MPR? Bovine CI-MPR d1–15 was immobilized to a sensor chip by amine coupling. (**A**) Mock- or phosphatase-treated recombinant tPA were injected at various concentrations. (**B**) Mock- or phosphatase-treated β-glucuronidase were injected at various concentrations. (**C**) At a concentration of 80 nM, mock-treated recombinant tPA was injected over the bovine CI-MPR d1–15 surface in the presence of 5 mM M6P, 5 mM G6P, or no inhibitor. (**D**) Same as in (**C**), but recombinant tPA was treated with phosphatase. Note that the M6P and G6P sensorgram curves overlay. *CI-MPR d1–15* cation-independent mannose 6-phosphate receptor domains 1–15, *tPA* tissue plasminogen activator, *M6P* mannose 6-phosphate, *G6P* glucose 6-phosphate.

We next aimed to determine the nature of the interaction between tPA and CI-MPR. The N-glycans of lysosomal enzymes are modified with M6P and M6P-GlcNAc, and CI-MPR binds these moieties though its carbohydrate recognition domains (d3, d5, d9, and d15). Because tPA is a glycoprotein that houses three N-linked glycosylation sites, we asked whether these glycans may contain M6P, allowing tPA to bind to CI-MPR in a M6P-dependent fashion. Recombinant tPA was treated with phosphatase and injected over an immobilized bovine CI-MPR d1–15 sensor chip. Compared to mock-treated tPA, phosphatase-treated tPA resulted in sensorgrams with diminished responses (Fig. 2A). We used β-glucuronidase as a positive control because this lysosomal enzyme is known to interact with CI-MPR via M6P on its N-linked glycans. Phosphatase-treated β-glucuronidase also resulted in diminished responses compared to mock-treated β-glucuronidase (Fig. 2B), but to a greater degree than was observed for phosphatase-treated tPA. Possible reasons for the residual response of phosphatase-treated tPA and β-glucuronidase include incomplete phosphatase digestion as well as the presence of M6P-GlcNAc, which is resistant to phosphatase digestion. Because tPA is also known to bind aggregated proteins^5^, immobilized CI-MPR aggregates may also be responsible for the residual responses of phosphatase-treated tPA.

To further investigate the interaction between tPA and CI-MPR, we incubated mock-treated tPA (Fig. 2C) and phosphatase-treated tPA (Fig. 2D) with a small molecule inhibitor, M6P, or a non-specific control, glucose 6-phosphate (G6P), prior to injection over immobilized bovine CI-MPR d1–15. Glucose is the C-2 epimer of mannose, and thus G6P serves as a control to demonstrate CI-MPR binding specificity for the axial 2-hydroxyl group of mannose. With bovine CI-MPR d1–15 immobilized, mock-treated recombinant tPA incubated with M6P resulted in a 55% decrease in average response in the final 10 s of tPA injection (Fig. 2C, green curve) while incubation with G6P resulted in a 26% decrease in average response (Fig. 2C, blue curve). When phosphatase-treated recombinant tPA was incubated with M6P or G6P, 13% and 12% decreases in response over the bovine CI-MPR d1–15 surface were observed, respectively (Fig. 2D, green and blue curves are overlaid). These data demonstrate that M6P has an inhibitory effect of greater than two-fold that of G6P, but this difference is eliminated upon phosphatase treatment. Together, these phosphatase experiments suggest that a M6P residue(s) on tPA are partially responsible for its binding to CI-MPR.

### M6P disrupts tPA and CI-MPR binding in a dose dependent manner

**Figure 3:**
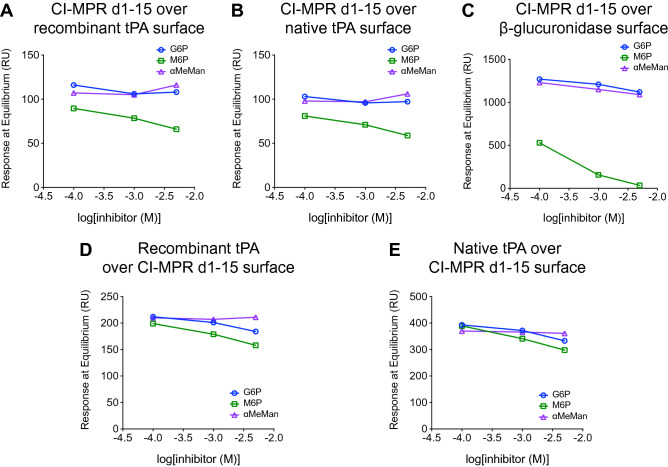
The interaction between CI-MPR and tPA is partially inhibited by M6P in a dose-dependent manner. (**A**–**C**) Bovine CI-MPR d1–15 at 20 nM was incubated with small molecule inhibitors, M6P, G6P, and αMeMan, at increasing concentrations (0.1, 1.0, and 5.0 mM) and flowed over various sensor chip surfaces. The response at equilibrium is plotted with respect to the log of the inhibitor concentration. (**A**) Recombinant tPA was immobilized by amine coupling. (**B**) Native tPA was immobilized by amine coupling. (**C**) β-Glucuronidase was immobilized by amine coupling. (**D**,**E**) Human CI-MPR d1–15 was immobilized to a sensor chip by amine coupling. Incubated in the presence of small molecule inhibitors at increasing concentrations (0.1, 1.0, and 5.0 mM), 40 nM tPA was flowed over the CI-MPR surface: (**D**) recombinant tPA in the presence of M6P, G6P, and αMeMan; (**E**) native tPA in the presence of M6P, G6P, and αMeMan. *CI-MPR d1–15* cation-independent mannose 6-phosphate receptor domains 1–15, *tPA* tissue plasminogen activator, *M6P* mannose 6-phosphate, *G6P* glucose 6-phosphate, *αMeMan* α-methyl mannoside.

We investigated the dose effect of the small molecule inhibitor, M6P, in SPR studies. G6P and α-methyl mannoside (αMeMan) were used as controls in these experiments. To further validate our findings, we performed these experiments with two different, but complementary, configurations: CI-MPR d1–15 injected over a tPA surface and tPA injected over a CI-MPR d1–15 surface. Bovine CI-MPR d1–15 was preincubated with M6P, G6P, and αMeMan at three concentrations: 0.1, 1.0, and 5.0 mM. This preincubation step enabled binding site saturation with these small molecules. The resulting mixture was then injected over recombinant tPA (Fig. 3A) and native tPA (Fig. 3B) surfaces, and a dose-dependent decrease in signal was observed with increasing concentrations of M6P, but not with G6P or αMeMan. As a positive control, bovine CI-MPR d1–15 was injected over a surface with β-glucuronidase immobilized. A dose-dependent decrease in equilibrium response was also observed with increasing concentrations of M6P, and almost complete inhibition was observed with 5.0 mM M6P (Fig. 3C). This finding is similar to the experiments above, where greater inhibition in binding was observed with phosphatase-treated β-glucuronidase compared to phosphatase-treated tPA (Fig. 2A,B).

In the reciprocal set-up, human CI-MPR d1–15 was immobilized to a sensor chip. Recombinant tPA (Fig. 3D) and native tPA (Fig. 3E) were incubated with M6P, G6P, or αMeMan and then injected over this surface. The greatest dose dependent decrease in equilibrium response was observed with M6P compared to G6P and αMeMan. The experiments with CI-MPR immobilized may result in less robust M6P inhibition because immobilized CI-MPR is unable to be pre-incubated with M6P unlike when CI-MPR is injected. Nonetheless, these data demonstrate that M6P disrupts binding between tPA and CI-MPR in a dose-dependent manner.

### The five N-terminal extracellular domains of CI-MPR are sufficient for tPA binding

**Figure 4:**
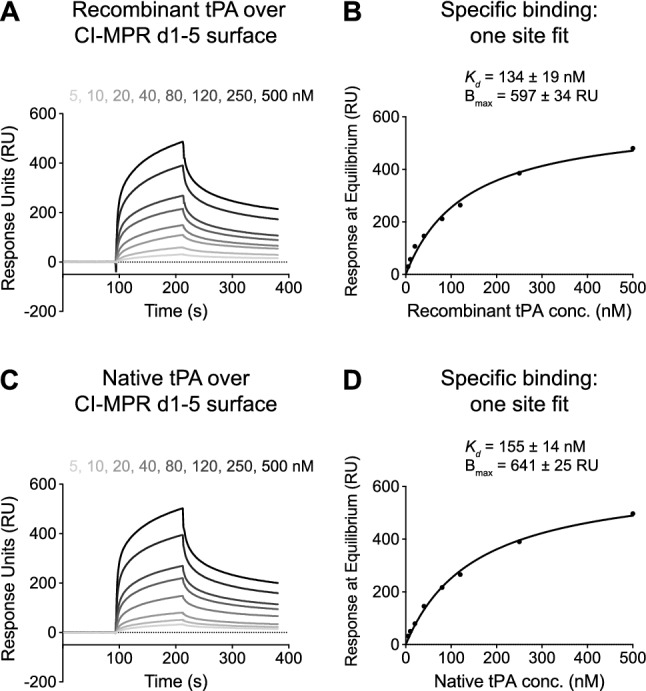
Recombinant and native tPA bind to the N-terminal third of CI-MPR with similar kinetics. Human CI-MPR d1-5 (containing a C-terminal biotin) was immobilized to a streptavidin sensor chip. (**A**) Recombinant tPA was injected at various concentrations. (**B**) For each sensorgram in (**A**), the response at equilibrium is plotted with respect to recombinant tPA concentration. The data are fit using a one site specific binding model. *K*_*d*_ and B_max_ are reported above the curve (best fit value ± SEM). (**C**,**D**) Same as in (**A** and **B**, respectively), but with native tPA. *CI-MPR d1-5* cation-independent mannose 6-phosphate receptor domains 1–5, *tPA *tissue plasminogen activator.

We next sought to more specifically map the region of CI-MPR that interacts with tPA. Because the related molecule, plasminogen, binds the N-terminus of CI-MPR, we investigated whether this is also true for tPA. A construct containing the five N-terminal domains of human CI-MPR (CI-MPR d1-5) was expressed, biotinylated, and immobilized to a streptavidin chip. Recombinant tPA was injected over the CI-MPR d1–5 surface at increasing concentrations, and responses were observed with each sample (Fig. 4A). These responses at equilibrium were plotted with respect to tPA concentration, and the data were fit with a one site specific binding model (Fig. 4B). The *K*_*d*_ was calculated to be 134 ± 19 nM. We also performed these experiments with native tPA. Comparable sensorgrams were observed when native tPA was injected over the CI-MPR d1-5 chip (Fig. 4C). The results from the one site specific binding fit yielded similar results, where the *K*_*d*_ was calculated to be 155 ± 14 nM (Fig. 4D). In summary, both recombinant and native tPA are able to bind to the five N-terminal domains of CI-MPR with similar affinities compared to CI-MPR d1–15.

### M6P partially disrupts binding between tPA and CI-MPR d1–5

**Figure 5:**
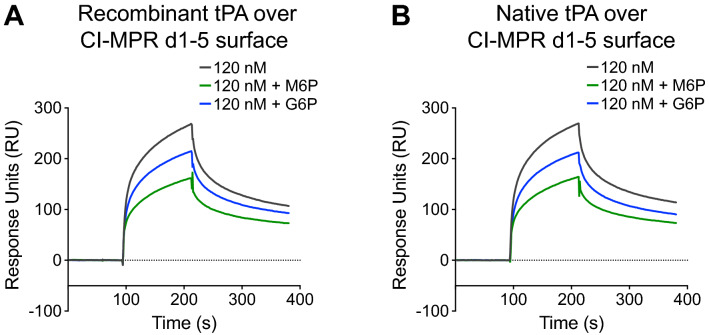
tPA binding to immobilized CI-MPR d1-5 is partially inhibited by M6P. (**A**) C-terminal biotinylated human CI-MPR d1-5 was immobilized to a streptavidin sensor chip, and recombinant tPA was injected at 120 nM in the presence of 5 mM M6P or 5 mM G6P. The resulting sensorgrams are shown. (**B**) Same as in (**A**), but with native tPA. *CI-MPR d1-5* cation-independent mannose 6-phosphate receptor domains 1–5, *tPA* tissue plasminogen activator, *M6P* mannose 6-phosphate, *G6P* glucose 6-phosphate.

We next tested whether tPA bound to immobilized CI-MPR d1–5 in a M6P-dependent manner. With CI-MPR d1–5 immobilized, recombinant tPA incubated with 5 mM M6P was injected, and a 40% decrease in response at equilibrium was observed (Fig. 5A). When recombinant tPA was injected in the presence of 5 mM G6P, only a 20% decrease in equilibrium response was observed (Fig. 5A). Native tPA was also injected over immobilized CI-MPR d1-5 with the same concentrations of M6P and G6P, and equilibrium responses decreased by 39% and 21%, respectively (Fig. 5B). Thus, with regard to the interaction between two tPA sources and immobilized CI-MPR d1-5, the inhibitory effect of M6P is approximately two-fold greater than the inhibitory effect with G6P, similar to the findings with CI-MPR d1–15 (Fig. 2C). Our previous SPR experiments have shown that binding between M6P-containing lysosomal enzymes and CI-MPR is completely inhibited by M6P, but only slightly inhibited by G6P^24,25,27^. Therefore, the observation that M6P inhibits binding by ~ 40%, with G6P only inhibiting binding by ~ 20%, suggests that the interaction between tPA and CI-MPR d1-5 is at least partially mediated by M6P.

### N-linked glycans containing phosphate groups are observed at tPA N448

The SPR experiments suggested that at least a fraction of tPA is modified with M6P, allowing it to bind the carbohydrate recognition sites of CI-MPR. However, the possibility remained that tPA binding to CI-MPR d1-5 is through a protein–protein interaction (i.e., not through M6P) and that the observed M6P inhibition is indirect. For example, upon binding to a carbohydrate recognition domain, M6P may induce a conformational change in CI-MPR, making it no longer amenable to tPA binding. In order to determine if tPA is actually modified with M6P, reversed-phase liquid chromatography-mass spectrometry analyses were performed on peptides and glycopeptides derived from recombinant and native tPA.

The amino acid sequence of tPA glycoprotein contains three consensus N-linked glycosylation sites (N-X-S/T) at N117, N184 and N448. In order to enhance sequence coverage and peptide detectability, recombinant and native tPA glycoproteins were digested following three conditions: chymotrypsin, chymotrypsin/Glu-C, or trypsin/Glu-C. We subjected peptide/glycopeptide mixtures produced by these conditions to LC–MS^*n*^ analysis and mapped the presence of phosphorylated glycans on tPA glycopeptides. Among the three predicted N-linked glycosylation sites, N117 glycosylated peptides were only detected following trypsin/Glu-C or chymotrypsin/Glu-C digestion, whereas N184 and N448 glycosylated peptides were only detected following trypsin/Glu-C or chymotrypsin alone digestion (Supplementary table 1, Supplementary figure 1).

**Figure 6:**
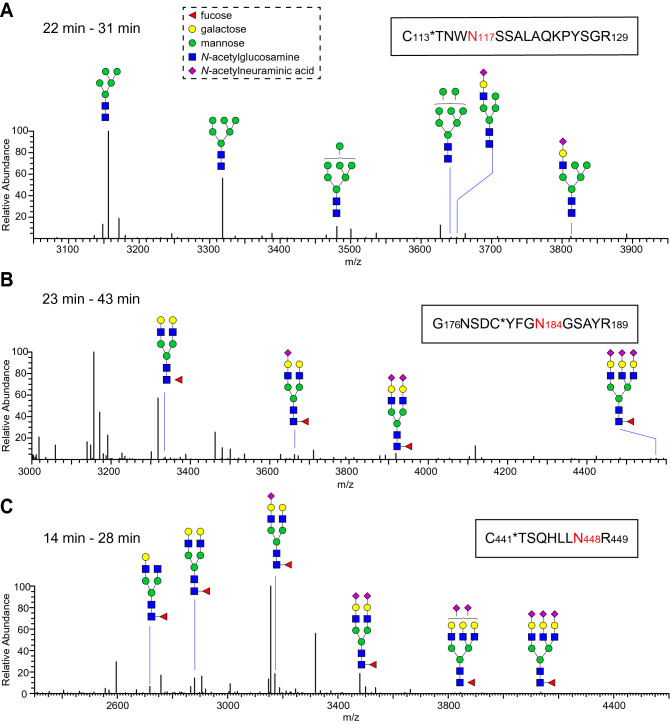
Major N-glycan species detected at the three glycosylation sites of native tPA. Native tPA was digested with trypsin and endoproteinase GluC to obtain glycopeptides (N-glycosylation site highlighted red in sequence). Full MS spectra were averaged across the time region in which the target glycopeptides eluted during the LC–MS run and deconvoluted for charge state. The glycoform profile for the three N-glycosylation sites of tPA are shown: N117 (**A**), N184 (**B**), N448 (**C**). The N117 site carries primarily high-mannose and hybrid type glycans, while complex N-glycans were most abundant at the N184 and N448 sites. Monosaccharides are represented using the Symbol Nomenclature for Glycans [dashed box in panel (A)]. See Supplementary figure 2 for the peptide glycoforms detected on recombinant tPA.

**Figure 7:**
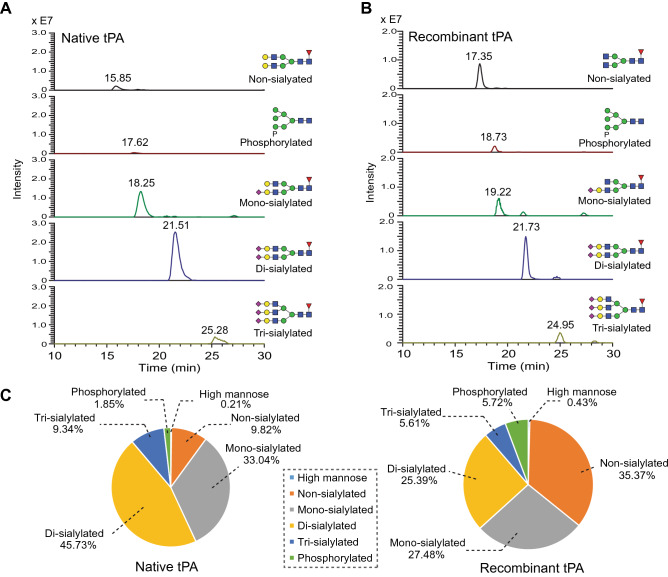
Glycopeptide profiles at N448 from native or recombinant tPA. Native or recombinant tPA glycopeptides obtained from trypsin/endoproteinase Glu C digestion were analyzed by reversed-phase (C18) LC–MS/MS. (**A**,**B**) Extracted ion chromatograms presenting the presence of the indicated peptide glycoform for (**A**) native and (**B**) recombinant tPA. (**C**) Pie charts present the relative abundance of each class of glycopeptide glycoform at the N448 site.

Glycoproteomic analysis revealed that both recombinant and native tPA carry primarily high-mannose and hybrid type glycans at N117, while complex type N-glycans were most abundant at N184 and N448 (Fig. 6, Supplementary figure 2). N448 glycopeptides carry glycans that are more completely sialylated on native tPA than on recombinant tPA; 55% of the native tPA peptide glycoforms were detected as di-sialylated biantennary or tri-sialylated triantennary glycoconjugates whereas these glycoforms accounted for only 31% of the glycoforms for recombinant tPA (Fig. 7, Supplementary table 2). Consistent with this disparity in sialylation, 43% of the glycopeptide glycoforms were undersialylated in the native tPA compared to 63% for the recombinant protein. This distribution of glycoforms was consistent across all the digests analyzed.

**Figure 8:**
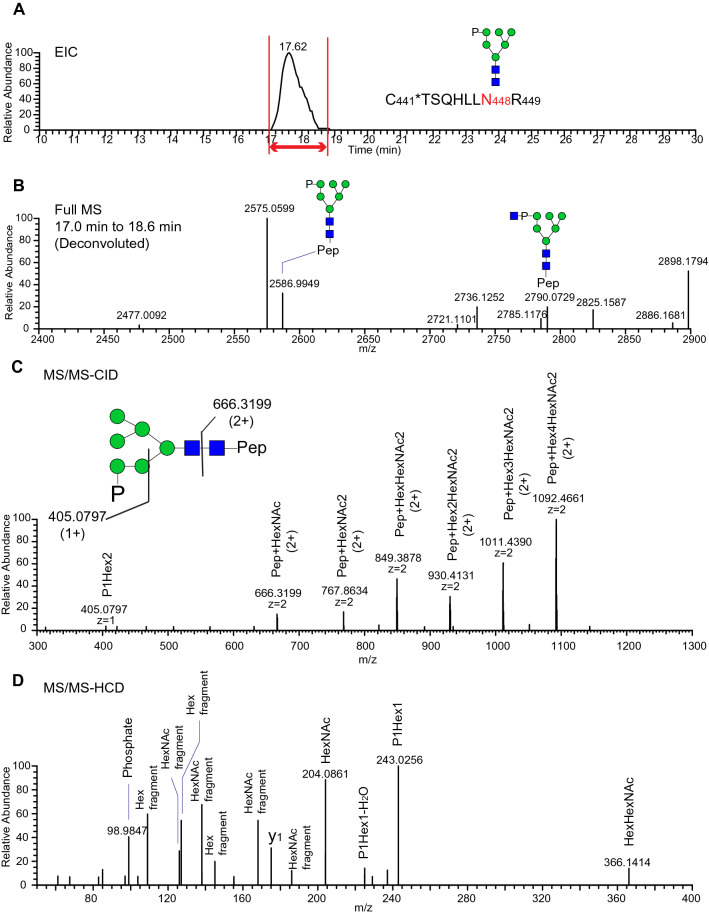
M6P is detected at tPA N448. (**A**) Extracted ion chromatogram of native tPA N448 glycopeptide carrying phosphorylated, high mannose glycoform (P1M6N2). (**B**) Deconvoluted, time-averaged full mass spectrum of glycopeptides eluting between 17 to 18.6 min. (**C**) Tandem mass spectrum (MS/MS) collision-induced dissociation of glycopeptide showing series of neutral losses indicating high-mannose type glycan structure as well as fragment ion at m/z 405. 0797, corresponding to a P1Hex2 fragment. (**D**) MS/MS higher collision energy dissociation (HCD) of glycopeptide in (**C**) reveals fragment ions for phosphate (m/z 98.9847) and P1Hex1 (m/z 243.0256).

N-glycans containing M6P were detected at N448 on both recombinant and native tPA (Fig. 7, Supplementary table 2) but at signal intensities lower than for peptides carrying complex type glycans at this site (Fig. 7, Supplementary table 2). Glycopeptides containing the N448 glycosylation site were detected as glycoforms containing phosphate monoesters (M6P) and as GlcNAc-covered diesters (GlcNAc-P-Man) (Fig. 8A,B). MS/MS analysis of phospho-glycopeptides confirmed the presence of phosphorylated hexose and phosphate fragment ions (Fig. 8). For example, the m/z 405.0797 ion in the MS/MS-CID spectrum (Fig. 8C) corresponds to an oxonium ion of phosphate1hexose2 (theoretical m/z 405.0791). Additionally, the m/z 98.9847 and m/z 243.0256 ions in the HCD spectrum (Fig. 8D) correspond to phosphoric acid ion, H_4_PO_4_^+^ (theoretical m/z 98.9842) and oxonium ion of phosphate1hexose1 (theoretical m/z 243.0264), respectively. Based on the detection of signature phospho-glycan signature ions, LC–MS/MS glycoproteomic analysis identified three phospho-glycoforms in native tPA, and four in recombinant tPA at the N448 glycosylation site (Fig. 7, Supplementary table 2).

## Discussion

In addition to lysosomal enzymes, several other molecules are modified with M6P on their N-linked glycans. Some examples include DNase I^28^, TGF-β latency-associated peptide^29^, and leukemia inhibitory factor (LIF)^30^. Glycans on some viruses, such as herpes simplex virus^31^ and varicella zoster virus^32^, have also been reported to contain M6P. In this report, we added to this list by directly measuring the existence of tPA glycoforms containing M6P by mass spectrometry. We also demonstrated that tPA binds to the extracellular region and the five N-terminal domains of CI-MPR (CI-MPR d1–15 and CI-MPR d1-5, respectively) by SPR and showed that this binding is partially inhibited by M6P and phosphatase treatment. While we identified tPA as a new CI-MPR ligand, future experiments are required to ascertain the physiological relevance of this interaction.

tPA is an FDA-approved, broadly used biotherapeutic. Thus, its N-glycosylation has been extensively characterized^33–37^. Previous studies have described glycan heterogeneity at each of the N-linked glycosylation sites of tPA but have not reported phosphoglycans^37^. Unless analytic efforts are focused on detecting phosphoglycoforms, they can be missed. We detect similar distributions of complex, sialylated bi- and tri-antennary glycans at tPA glycosylation sites as described previously but add the presence of phosphomonoester/phosphodiester-containing glycans at N448. Phosphorylated glycans and phosphorylated glycoconjugates (including glycopeptides) generally exhibit greatly reduced ionization efficiency compared to their non-phosphorylated counterparts, impairing detection sensitivity by nano- or electro-spray ionization interfaces. Based on the SPR results described here, we focused specifically on determining whether phosphoglycoforms of tPA were present. Targeted manual interpretation of LC–MS/MS data captured precursor and fragmentation signatures that unambiguously detected M6P and GlcNAc-P-Man modifications at the N448 glycosylation site of native and recombinant tPA. While we report that the relative abundance of phosphorylated glycans at N448 is 1.85% for native tPA and 5.72% for recombinant tPA, we acknowledge that these values likely under-represent their actual prevalence due to inefficient ionization.

Because the relative abundance of phosphorylated glycans compared to total glycans on tPA is low, the effective concentration of tPA capable of binding CI-MPR is probably significantly lower than total tPA concentration. This would result in corresponding increases in apparent affinities reported in this study. For example, the calculated *K*_*d*_ values of recombinant and native tPA over the human CI-MPR d1–15 surface were found to be 23 and 81 nM, respectively (Fig. 1A,B). Factoring in the stoichiometry of tPA that contains phosphorylated mannose (2–6%), the *K*_*d*_ values are possibly closer to 0.5–5.0 nM (i.e., 17 to 50-fold lower than 23–81 nM). This range is quite similar to the 1–5 nM affinity range between phosphorylated lysosomal enzymes and CI-MPR reported by Sleat and Lobel^38^.

Although we observed that tPA consists of M6P-containing glycoforms, is there a biological role of M6P on tPA? Three explanations for the presence of M6P on tPA are discussed here. First, the presence of M6P on tPA may be an artifact of overexpression. Non-native and aberrant protein glycosylation is often observed in cancer cells and transfected cells expressing recombinant proteins used for the generation of biotherapeutics^39–42^. Thus, by overwhelming expression machinery, a fraction of tPA may be incorrectly phosphorylated by the *N*-acetylglucosamine-1-phosphate transferase complex, which is the first step in the generation of the M6P moiety. As mentioned above, TGF-β latency associated peptide is reported to contain M6P^29^, and latency associated peptide binding to CI-MPR was reported as necessary for TGF-β activation^43^. However, a more recent study demonstrated: (1) M6P is undetectable on native latent TGB-β; (2) only ~ 3% of the N-glycans on CHO-produced latent TGF-β contain M6P; and (3) TGF-β activation in corneal cells is not M6P-dependent^44^. Thus, the authors of this study concluded that the M6P modification on TGF-β is unlikely to be physiologically relevant^44^. The amount of M6P on LIF also seems to be dependent on the cell line in which it is expressed. Blanchard et al. found that less than 5% of native LIF contains M6P but ~ 15% of CHO-produced LIF contains M6P^45^, and Barnes et al. found that CHO-produced LIF contains as much as 35–45% M6P^46^. Our findings that M6P-containing glycans are more abundant in recombinant (CHO-produced) tPA compared to native tPA are consistent with the pattern found in CHO-produced vs native latent TGF-β and LIF. Therefore, expression of recombinant proteins in certain cell lines, such as CHO cells, may lead to higher levels of mannose phosphorylation.

**Figure 9:**
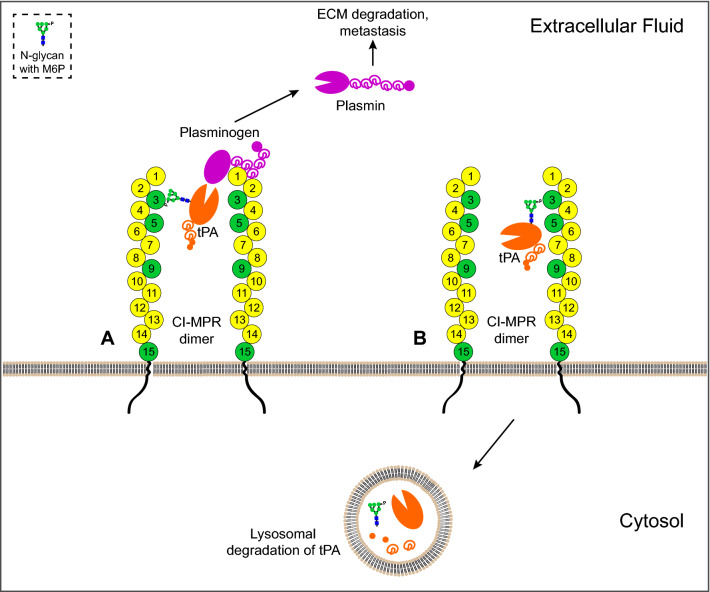
Models of possible biological significance of CI-MPR and tPA binding. CI-MPR domains that can bind M6P and/or M6P-GlcNAc are represented in green circles, while all other domains are represented in yellow circles. (**A**) CI-MPR may serve as a scaffold to bring tPA and plasminogen in close proximity to one another, which may facilitate plasmin generation. Active plasmin can degrade ECM and enable cancer cell metastasis. (**B**) Similar to M6P-containing lysosomal enzymes, the M6P or M6P-GlcNAc binding sites of CI-MPR may sequester tPA, thereby targeting tPA to the lysosome for degradation. In this model, tPA is shown bound to CI-MPR domain 3, but it is possible that tPA may bind to CI-MPR via its other carbohydrate recognition domains: domain 5, domain 9, or domain 15. *CI-MPR* cation-independent mannose 6-phosphate receptor, *tPA* tissue plasminogen activator, *M6P* mannose 6-phosphate, *GlcNAc* *N*-acetylglucosamine, *ECM* extracellular matrix.

Second, CI-MPR may function as a scaffold to facilitate plasmin generation by bringing plasminogen and tPA near each other as others have suggested with plasminogen and uPAR^21,22^. Plasminogen binds to the N-terminus of CI-MPR^14,15^, and in this report, we found that tPA binds the N-terminal third of CI-MPR in a M6P-dependent manner. Given the glycan specificity of the two carbohydrate-binding sites within the N-terminal region of CI-MPR (domain 3 recognizes phosphate monoesters while domain 5 recognizes phosphate diesters)^27^, tPA bearing phosphate monoesters (M6P) would interact with domain 3 whereas tPA bearing GlcNAc-covered diesters (GlcNAc-P-Man) would interact with domain 5. Because CI-MPR exists as a dimer in the plasma membrane, plasminogen and tPA can be brought together by each binding to a CI-MPR monomer (Fig. 9A). Still the question remains on whether the scaffold hypothesis has a biological role. Because CI-MPR is generally viewed as a tumor suppressor^47,48^, the model that CI-MPR promotes plasminogen activation for metastasis is conflicting. Alternatively, CI-MPR is highly expressed in the central nervous system (reviewed in^49^), as is tPA (reviewed in^6^). CI-MPR has also been shown to play a role in memory consolidation and enhancement^50,51^. Thus, CI-MPR may modulate tPA activity in neuronal processes, such as in learning and memory.

Third, M6P on tPA N-glycans may play a role in tPA clearance. CI-MPR captures incorrectly secreted lysosomal enzymes modified with M6P, which allows for their return to the lysosome. Another major CI-MPR function is to downregulate extracellular IGF2, and thus, the possibility that CI-MPR also downregulates tPA is consistent with this growth suppressor role of CI-MPR. The N-glycan structures on tPA are known to significantly influence its turnover^52^, and lectins on the surface of liver cells, such as mannose and galactose receptors, have been shown to endocytose tPA^53^. Thus, CI-MPR may also play a role in tPA clearance. In this model, phosphorylated high mannose structures on tPA may bind to any of the carbohydrate recognition domains on CI-MPR, which can traffic tPA to the lysosome for destruction (Fig. 9B). This is the case with leukemia inhibitory factor, a cytokine that contains M6P^30^, where CI-MPR mediates the internalization and lysosomal destruction of this cytokine^45^. Therefore, the possibility also exists that a cell may increase the M6P content on tPA as a mechanism to downregulate this serine protease as a means to maintain homeostasis of the plasminogen activation system.

The second and third explanations above must be considered in the context that only 2–6% of tPA glycans contain phosphorylated mannose. Therefore, these projected roles would only apply to a fraction of tPA. While the biological roles of the interaction between CI-MPR and tPA are still unknown, we have identified that these two molecules bind in a M6P-dependent manner, and we directly demonstrated that tPA consists of M6P-containing glycoforms. Future studies are needed on whether this interaction occurs physiologically, and if so, whether CI-MPR enhances or downregulates the function of tPA. It is possible that the role of CI-MPR on tPA biology is cell-type specific, such as in the central nervous system where both proteins are highly expressed. To our knowledge, our study is the first to demonstrate that tPA is a ligand for CI-MPR, further highlighting the role of CI-MPR in the plasminogen activation system.

## Supplementary Information

Supplementary Information.

## Data Availability

The datasets generated during in this study are available from the corresponding author on reasonable request.
