# Supplementary material for: Tissue plasminogen activator is a ligand of cation-independent mannose 6-phosphate receptor and consists of glycoforms that contain mannose 6-phosphate
Source: Sci Rep. 2021 Apr 15;11:8213. doi: 10.1038/s41598-021-87579-z (PMC8050316; doi:10.1038/s41598-021-87579-z)
Supplement: Supplementary file 1 — Supplementary Information. [file 41598_2021_87579_MOESM1_ESM.pdf]

Supplemental information

Tissue plasminogen activator is a ligand of cation-independent mannose 6-phosphate receptor and consists of glycoforms that contain mannose 6-phosphate

James J. Miller[1], Richard N. Bohnsack[1], Linda J. Olson[1], Mayumi Ishihara[2], Kazuhiro Aoki[2], Michael Tiemeyer[2]*, Nancy M. Dahms[1]*

[1]Department of Biochemistry, Medical College of Wisconsin, Milwaukee, WI

[2]Complex Carbohydrate Research Center, University of Georgia, Athens, GA

*Correspondence: Nancy M. Dahms, PhD, Department of Biochemistry, Medical College of Wisconsin, 8701 W. Watertown Plank Rd., Milwaukee, WI 53226, Phone: 414-955-4698, Fax: 414-955-6510, Email: ndahms@mcw.edu, ORCID identifier: https://orcid.org/0000-0001-6819-0590 or Michael Tiemeyer, PhD, Complex Carbohydrate Research Center, 315 Riverbend Rd, University of Georgia, Athens, GA 30602, Phone: 706-542-2740, Fax: 706-542-4412, Email: mtiemeyer@ccrc.uga.edu, ORCID identifier: https://orcid.org/0000-0002-8704-9143

Chymotrypsin

1 SYQVICRDEK TQMIYQQHQS WLRPVLRSNR VEYCWCNSGR AQCHSVPVKS CSEPRCFNGG TCQQALYFSD FVCQCPEGFA

81 GKCCEIDTRA TCYEDQGISY RGTWSTAESG AECTNWNSSA LAQKPYSGRR PDAIRLGLGN HNYCRNPDRD SKPWCYVFKA

161 GKYSSEFCST PACSEGNSDC YFGNGSAYRG THSLTESGAS CLPWNSMILI GKVYTAQNPS AQALGLGKHN YCRNPDGDAK

241 PWCHVLKNRR LTWEYCDVPS CSTCGLRQYS QPQFRIKGGL FADIASHPWQ AAIFAKHRRS PGERFLCGGI LISSCWILSA

321 AHCFQERFPP HHLTVILGRT YRVVPGEEEQ KFEVEKYIVH KEFDDDTYDN DIALLQLKSD SSRCAQESSV VRTVCLPPAD

401 LQLPDWTECE LSGYGKHEAL SPFYSERLKE AHVRLYPSSR CTSQHLLNRT VTDNMLCAGD TRSGGPQANL HDACQGDSGG

481 PLVCLNDGRM TLVGIISWGL GCGQKDVPGV YTKVTNYLDW IRDNMRP

Chymotrypsin/Glu-C (DE)

1 SYQVICRDEK TQMIYQQHQS WLRPVLRSNR VEYCWCNSGR AQCHSVPVKS CSEPRCFNGG TCQQALYFSD FVCQCPEGFA

81 GKCCEIDTRA TCYEDQGISY RGTWSTAESG AECTNWNSSA LAQKPYSGRR PDAIRLGLGN HNYCRNPDRD SKPWCYVFKA

161 GKYSSEFCST PACSEGNSDC YFGNGSAYRG THSLTESGAS CLPWNSMILI GKVYTAQNPS AQALGLGKHN YCRNPDGDAK

241 PWCHVLKNRR LTWEYCDVPS CSTCGLRQYS QPQFRIKGGL FADIASHPWQ AAIFAKHRRS PGERFLCGGI LISSCWILSA

321 AHCFQERFPP HHLTVILGRT YRVVPGEEEQ KFEVEKYIVH KEFDDDTYDN DIALLQLKSD SSRCAQESSV VRTVCLPPAD

401 LQLPDWTECE LSGYGKHEAL SPFYSERLKE AHVRLYPSSR CTSQHLLNRT VTDNMLCAGD TRSGGPQANL HDACQGDSGG

481 PLVCLNDGRM TLVGIISWGL GCGQKDVPGV YTKVTNYLDW IRDNMRP

Trypsin/Glu-C (DE)

1 SYQVICRDEK TQMIYQQHQS WLRPVLRSNR VEYCWCNSGR AQCHSVPVKS CSEPRCFNGG TCQQALYFSD FVCQCPEGFA

81 GKCCEIDTRA TCYEDQGISY RGTWSTAESG AECTNWNSSA LAQKPYSGRR PDAIRLGLGN HNYCRNPDRD SKPWCYVFKA

161 GKYSSEFCST PACSEGNSDC YFGNGSAYRG THSLTESGAS CLPWNSMILI GKVYTAQNPS AQALGLGKHN YCRNPDGDAK

241 PWCHVLKNRR LTWEYCDVPS CSTCGLRQYS QPQFRIKGGL FADIASHPWQ AAIFAKHRRS PGERFLCGGI LISSCWILSA

321 AHCFQERFPP HHLTVILGRT YRVVPGEEEQ KFEVEKYIVH KEFDDDTYDN DIALLQLKSD SSRCAQESSV VRTVCLPPAD

401 LQLPDWTECE LSGYGKHEAL SPFYSERLKE AHVRLYPSSR CTSQHLLNRT VTDNMLCAGD TRSGGPQANL HDACQGDSGG

481 PLVCLNDGRM TLVGIISWGL GCGQKDVPGV YTKVTNYLDW IRDNMRP

Supplementary figure 1: Cleavage sites of proteases used for the glycopeptide mapping of tPA by MS. Recombinant and native tPA were digested with three different enzyme combinations (chymotrypsin alone, chymotrypsin with Glu-C, and trypsin with Glu-C) to enhance the production of informative glycopeptides. The predicted cleavage sites of each protease are shown underlined. The sites of N-glycosylation are presented with red font. The glycopeptides detected from each digest are highlighted with yellow.

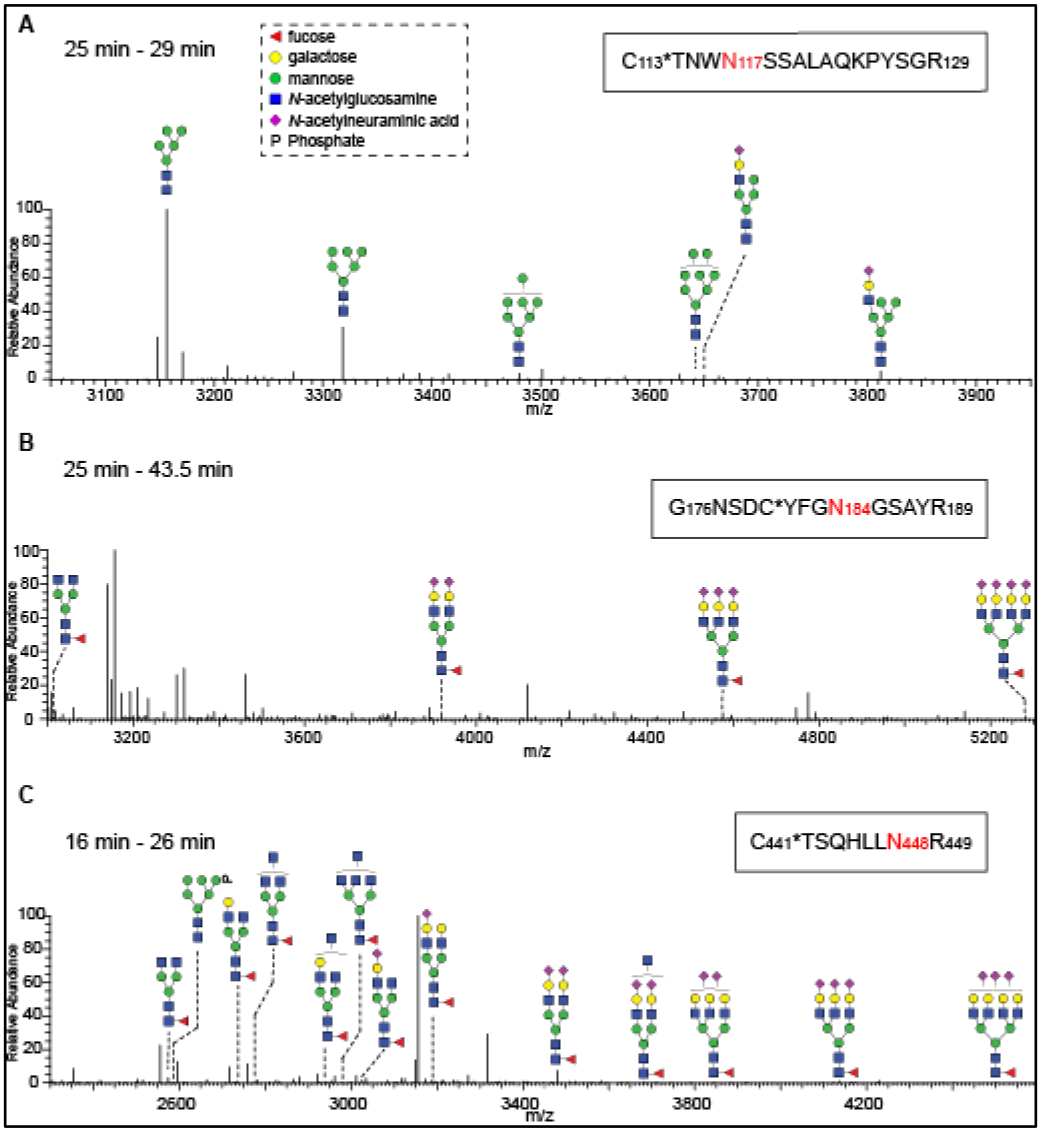

Supplementary figure 2: Major N-glycan species detected at the three glycosylation sites of recombinant tPA. Recombinant tPA was digested with trypsin and endoproteinase GluC to obtain glycopeptides (N-glycosylation site highlighted red in sequence). Full MS spectra were averaged across the time region in which the target glycopeptides eluted during the LC-MS run and deconvoluted for charge state. The glycoform profile for the three N-glycosylation sites of tPA are shown: N117 (**A**), N184 (**B**), N448 (**C**). Monosaccharides are represented using the Symbol Nomenclature for Glycans (dashed box in panel (**A**)).

Supplementary table 1:

List of glycopeptides detected from native and recombinant tPA by MS.

Site of glycosylation	Proteases used	Position	Sequence
N117	Chymotrypsin/Glu-C	113-126	C_{113}*TNWN_{117}@SSALAQKPY_{126}
	Trypsin/Glu-C	113-129	C_{113}*TNWN_{117}@SSALAQKPYSGR_{129}
N184	Chymotrypsin	182-188	F_{182}GN_{184}@GSAY_{188}
	Trypsin/Glu-C	176-189	G_{176}NSDC*YFGN_{184}@GSAYR_{189}
N448	Chymotrypsin	448-456	N_{448}@RTVTDNML_{456}
	Trypsin/Glu-C	441-449	C_{441}*TSQHLLN_{448}@R_{449}

C*: Carbamidomethylated cysteine; N@: site of N-glycosylation

Supplementary table 2:

Glycan compositions detected at N448 of native and recombinant tPA.

Glycoform	Peak intensity		Peak intensity %	
	Native tPA	Recombinant tPA	Native tPA	Recombinant tPA
M5N2	1.37E+05	4.31E+05	0.21%	0.43%
M3N2F	2.35E+04	2.78E+05	0.04%	0.28%
NM3N2F	3.78E+05	3.58E+06	0.59%	3.54%
N2M3N2F	7.51E+05	1.27E+07	1.17%	12.57%
GalN2M3N2F	1.08E+06	4.60E+06	1.68%	4.55%
N3M2N2F	2.76E+05	4.35E+06	0.43%	4.31%
Gal2N2M3N2F	2.90E+06	2.97E+06	4.51%	2.94%
GalN3M3N2F	2.42E+05	1.96E+06	0.38%	1.94%
N4M3N2F	7.22E+04	2.71E+06	0.11%	2.68%
Gal2N3M3N2F	2.46E+05	6.00E+05	0.38%	0.59%
GalN4M3N2F	5.72E+04	1.66E+06	0.09%	1.64%
Gal3N3M3N2F	2.83E+05	3.21E+05	0.44%	0.32%
NeuAc1Gal1N2M3N2F	3.70E+06	1.25E+07	5.76%	12.37%
NeuAc1Gal2N2M3N2F	1.43E+07	8.03E+06	22.26%	7.95%
NeuAc1Gal1N3M3N2F	8.13E+05	2.22E+06	1.27%	2.20%
NeuAc1Gal2N3M3N2F	7.39E+05	1.34E+06	1.15%	1.33%
NeuAc1Gal1N4M3N2F	n.d.	2.25E+06	n.d.	2.23%
NeuAc1Gal3N3M2N2F	1.34E+06	5.77E+05	2.09%	0.57%
NeuAc1Gal2N4M3N2F	3.35E+05	8.42E+05	0.52%	0.83%
NeuAc2Gal2N2M3N2F	2.42E+07	1.70E+07	37.67%	16.83%
NeuAc2Gal2N3M3N2F	1.57E+06	2.10E+06	2.44%	2.08%
NeuAc2Gal3N3M3N2F	3.61E+06	2.70E+06	5.62%	2.67%
NeuAc2Gal2N4M3N2F	n.d.	1.28E+06	n.d.	1.27%
NeuAc2Gal3N4M3N2F	n.d.	2.57E+06	n.d.	2.54%
NeuAc3Gal3N3M2N2F	6.00E+06	3.16E+06	9.34%	3.13%
NeuAc3Gal3N4M3N2F	n.d.	1.78E+06	n.d.	1.76%
NeuAc3Gal4N4M3N2F	n.d.	7.26E+05	n.d.	0.72%
P1M5N2	2.18E+05	7.38E+05	0.34%	0.73%
P1M6N2	5.74E+05	2.28E+06	0.89%	2.26%
N1P1M6N2	3.97E+05	1.11E+06	0.62%	1.10%
P1NeuAc1Gal1N1M6N2F	n.d.	1.65E+06	n.d.	1.63%
	6.42E+07	1.01E+08	100.00%	100.00%

P: Phosphate; NeuAc: N-acetylneuraminic acid; Gal: Galactose; N: N-acetylglucosamine; M: Mannose; F: Fucose
